# Polyclonal on- and off-target resistance mutations in an *EML4-ALK* positive non-small cell lung cancer patient under ALK inhibition

**DOI:** 10.18632/oncotarget.28062

**Published:** 2021-09-14

**Authors:** Marcel Kemper, Georg Evers, Arik Bernard Schulze, Jan Sperveslage, Christoph Schülke, Georg Lenz, Thomas Herold, Wolfgang Hartmann, Hans-Ulrich Schildhaus, Annalen Bleckmann

**Affiliations:** ^1^Department of Medicine A, Hematology, Oncology, and Pneumology, University Hospital Muenster, 48149 Muenster, Germany; ^2^West German Cancer Center, Sites Muenster & Essen, 45147 Essen, Germany; ^3^Gerhard-Domagk-Institute of Pathology, University Hospital Muenster, 48149 Muenster, Germany; ^4^Institute of Clinical Radiology, University Hospital Muenster, 48149 Muenster, Germany; ^5^Institute of Pathology, University Hospital Essen, 45147 Essen, Germany; ^6^Department of Hematology/Medical Oncology, University Medical Center Goettingen, 37075 Goettingen, Germany; ^*^Authors share first authorship; ^#^Authors share last authorship

**Keywords:** NSCLC, resistance mutations, ALK, KRAS, ALK inhibitors

## Abstract

Treatment of advanced stage *anaplastic lymphoma kinase* (*ALK*) positive non-small cell lung cancer (NSCLC) with ALK tyrosine kinase inhibitors (TKIs) has been shown to be superior to standard platinum-based chemotherapy. However, secondary progress of disease frequently occurs under ALK inhibitor treatment. The clinical impact of re-biopsies for treatment decisions beyond secondary progress is, however, still under debate. Here, we report on two novel subsequent polyclonal on- and off-target resistance mutations in a patient with *ALK*-fused NSCLC under ALK inhibitor treatment. A 63-year-old male patient with an advanced stage *EML4-ALK* fused pulmonary adenocarcinoma was initially successfully treated with the second-generation ALK inhibitor alectinib and upon progressions subsequently with brigatinib, lorlatinib and chemoimmunotherapy (CIT). Progress to alectinib was associated with a so far undescribed *ALK* mutation (p.A1200_G1201delinsW) which was, however, tractable by brigatinib. An off-target *KRAS*-mutation (p.Q61K) occurred in association with subsequent progression under second-line TKI treatment. Third-line lorlatinib showed limited efficacy but chemoimmunotherapy resulted in disappearance of the *KRAS* mutant clone and clinical tumor control for another eight months. In conclusion, we suggest molecular profiling of progressive tumor disease also for *ALK*-positive NSCLC to personalize treatment in a subgroup of *ALK*-positive patients.

## INTRODUCTION

Despite new treatment options, metastatic non-small cell lung cancer (NSCLC) continues to be associated with the highest cancer-related mortality rates worldwide [1]. In addition to the growing landscape of immuno-oncology, a multitude of driver mutations and alterations in genes such as *EGFR* (epidermal growth factor receptor), *ALK* (anaplastic lymphoma kinase), *ROS1* (proto-oncogene tyrosine-protein kinase ROS1), *BRAF* (v-Raf murine sarcoma viral oncogene homolog B) and *NTRK1-3* (neurotrophic tyrosine kinase receptors 1–3) represent targets for personalized treatment options. Therefore, molecular testing at diagnosis and at relapse is essential in advanced NSCLC [2] and is even gaining importance in early tumor stages [3].

Rearrangement of the *ALK*-gene can be detected in 3–8% of NSCLC patients [4], with the *echinoderm microtubule-associated protein-like 4* (*EML4*) gene being the predominant genomic partner leading to the *EML4-ALK* fusion oncogene [5]. Aberrant ALK kinase activity can be specifically targeted with tyrosine kinase inhibitors (TKIs). The first-generation TKI crizotinib demonstrated superiority over standard platinum-based chemotherapy [6]. Subsequently, second- and third-generation ALK inhibitors such as alectinib [7], brigatinib [8] and lorlatinib [9] have shown increased benefit over crizotinib. Fourth-generation ALK inhibitors are currently being investigated [10]. However, the ALK-targeting approach loses effectiveness over time as on-target resistance mutations of *ALK* or *ALK* gene amplifications may occur which lead to disease progression [11–14].

Here, we report a novel *ALK*-exon 23 resistance mutation (p.A1200_G1201delinsW) that provided resistance to alectinib but was tractable by brigatinib. A subsequent off-target *KRAS*-exon 3 mutation (p.Q61K) was associated with secondary resistance to brigatinib but was basically sensitive to chemoimmunotherapy (CIT).

## CASE REPORT

A 63-year-old man with a ten pack-year history of tobacco smoking presented in our clinic with immobilizing lower back pain, drenching night sweats, and a weight loss of three kilograms over a few weeks. Radiography and magnetic resonance imaging (MRI) of the spine showed a pathologic fracture of lumbar vertebra 1 (L1) with intrusion into the spinal canal. Follow-up computed tomography (CT) revealed tumor mass consistent with lung cancer in the left upper pulmonary lobe, as well as metastatic lesions in mediastinal lymph nodes, the liver and the left adrenal gland. ^18^F-fluorodeoxyglucose positron emission tomography (FDG PET), CT, and cerebral MRI scans revealed additionally multiple osseous and brain metastases. Both the pathological examination of tissues from vertebral bone resection and from a lung biopsy revealed a cytokeratine 7/TTF1-positive pulmonary adenocarcinoma with low PD-L1 expression (tumor proportion score: 20%) as determined by immunohistochemistry. Molecular genetic analyses using fluorescence-*in-situ*-hybridization (FISH) and RNA sequencing provided evidence of *EML4-ALK* fusion variant 1 (exon 13/ exon 20), which is the most frequent (33%) variant in lung cancer [15].

**Figure 1:**
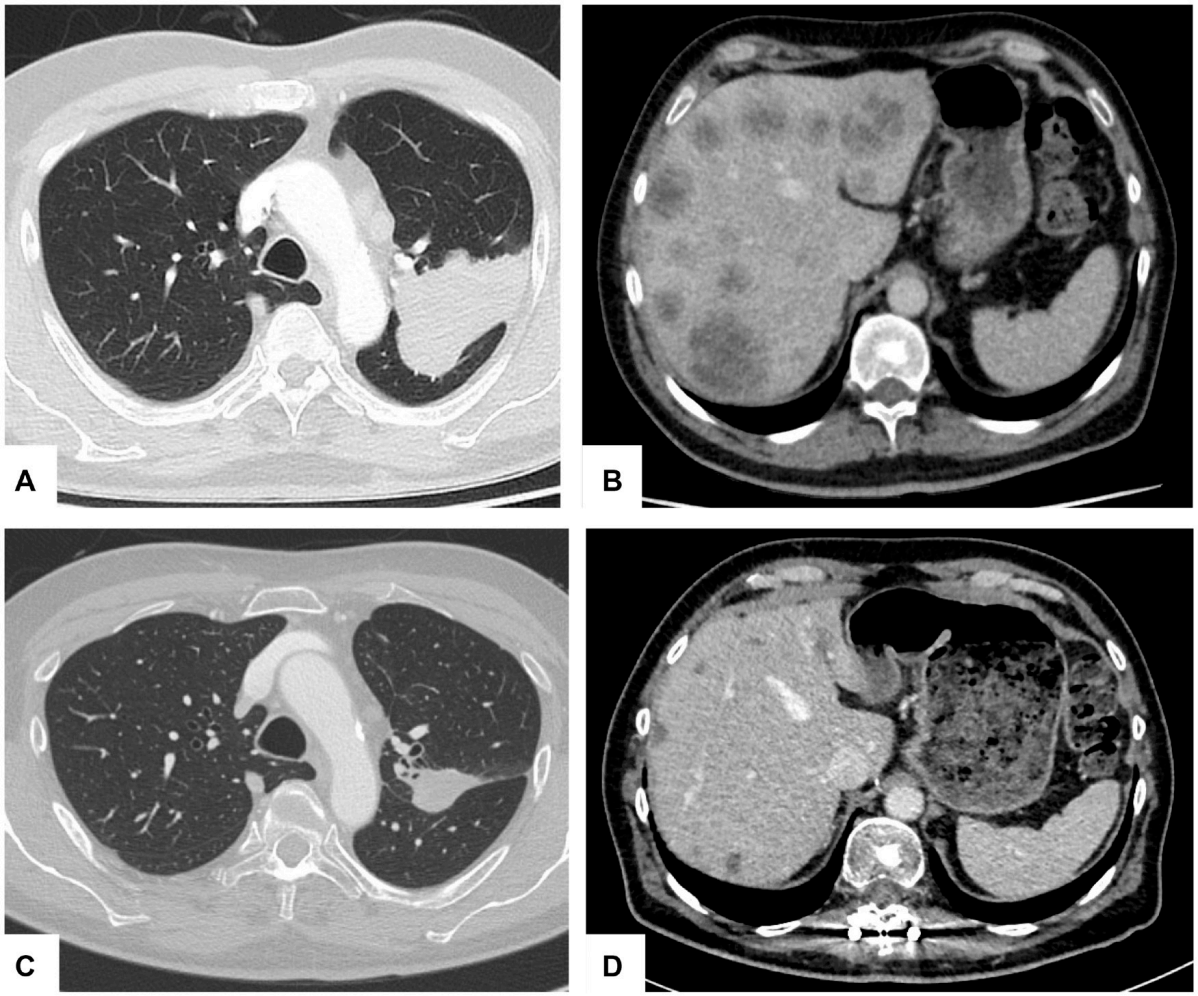
Hepatic and pulmonary tumor response during therapy. Axial computed tomography (CT) scans of left upper lobe pulmonary tumor and hepatic metastatic lesions at initial diagnosis (**A**, **B**) and after alectinib therapy ten months later (**C**, **D**) showing partial remission (PR).

**Figure 2:**
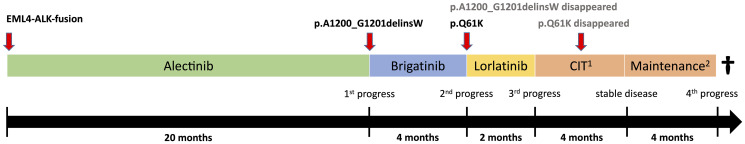
Therapy timeline and polyclonal tumor evolution. ^1^CIT: chemoimmuotherapy according to the Impower150 protocol with atezolizumab, carboplatin, paclitaxel and bevacizumab. ^2^Maintenance: Maintenance therapy with atezolizumab and bevacizumab.

First-line treatment with the second-generation TKI alectinib was initiated with concurrent stereotactic radiotherapy of the bone and brain lesions. Treatment resulted in partial remission (PR) for 20 months (Figure 1). At this timepoint worsening of clinical status occurred and a CT scan showed isolated secondary progression of hepatic metastases. Liquid biopsy and tissue re-biopsy of a liver metastasis independently yielded evidence of a previously undescribed *ALK*-exon 23 indel mutation (p.A1200_G1201delinsW) via next-generation sequencing (NGS) (Figure 2). At this time, the patient rejected the recommended treatment with lorlatinib because of its potential adverse neurocognitive effects. Therefore, a second-line treatment with brigatinib was started leading to relief of clinical symptoms and a stable disease for four months. Subsequently, tertiary progression of the hepatic metastases was noted while a further liquid biopsy and liver sampling both failed to demonstrate the previous *ALK* resistance mutation. However, the *EML4-ALK* fusion was still detected in the liver specimen. Interestingly, a so far undescribed activating *KRAS*-exon 3 mutation (p.Q61K) was identified in both the liquid and liver biopsy via NGS analysis (Figure 2). An inactivating *TP53* mutation (p.C238Y) was only found temporarily in a liquid biopsy but not in liver tissue at this time. Hence, therapy was switched to the third-generation TKI lorlatinib, which resulted in a stable disease for only two months. Due to further isolated hepatic disease progression the patient stopped TKI treatment and underwent combined chemoimmunotherapy and antiangiogenetic therapy according to the Impower150 protocol [16] based on discussion in a molecular tumor board at the West German Cancer Center. After four cycles of chemoimmunotherapy no further disease progression was observed, and the *KRAS* mutation disappeared from plasma as measured in a liquid biopsy. Hence, four cycles of maintenance therapy with atezolizumab and bevacizumab were conducted afterwards (Figure 2). Unfortunately, follow-up after four months revealed extensive hepatic and peritoneal disease progression, although cerebral MRI scans revealed no further evidence of previously detected brain metastases at all. Ultimately, rapid clinical deterioration resulted in patient’s death.

## DISCUSSION

On-target resistance mutations within the *ALK* gene have been shown to be common under treatment with second-generation ALK inhibitors such as alectinib [11]. There is a plethora of published data on preclinical characterization of these *ALK* mutations, and the activity of the common ALK-TKIs against these secondary mutations has been studied *in vitro* [11]. In contrast to this body of knowledge, molecular testing for *ALK* resistance mutations has not yet fully implemented in routine clinical practice. This is mainly due to the fact that second-line ALK-TKIs, namely brigatinib and lorlatinib, cover a nearly comparable spectrum of resistance mutations [11]. One exception is *ALK*-p.G1202R, which is the most common alteration after initial treatment with alectinib and which is thought to be more sensitive to lorlatinib than to brigatinib based on IC_50_ values [11, 13] and clinical data [12, 17]. However, there are no reports of any large-scale, head-to-head, randomized, controlled trials (RCT) in ALK-inhibitor-naïve NSCLC patients that compare currently the recommended dosages and administration frequency of the second-generation ALK inhibitor brigatinib with those of alectinib, which are currently both positioned as a first-line treatment option. Some of these questions are addressed by the ongoing prospective, randomized, open-label, phase II ABP study (NCT04318938). For the comparison of brigatinib and alectinib as second-line therapy after progression under crizotinib, the randomized, open label, phase III ALTA-3 study (NCT03596866) will add further information to this topic in future.

In this report, we describe a novel resistance mutation (*ALK*-p.A1200_G1201delinsW), which is located within the same genomic region encoding the kinase-domain. Based on our clinical findings from our patient we provide first evidence that i) this subtype of mutation confers resistance to alectinib as well and ii) brigatinib is obviously effective in this setting. We observed the disappearance of the ALK mutant clone and a clinical response of four months duration, which is basically in line with recently published progression free survival (PFS) data for brigatinib [18].

A tertiary progress of disease was induced in our patient by an unexpected off-target mutation, i.e., *KRAS*-p.Q61K. In contrast to other mutational subtypes [19, 20], the *KRAS*-p.Q61K mutation subtype has not yet been described as a resistance mutation in *ALK*-positive NSCLC but is known to be pathogenic from many other tumors [21, 22]. We found this specific *KRAS* mutation in both liquid biopsies and a liver tissue re-biopsy where the *EML4-ALK* fusion was still detectable. We decided to switch therapy to the third-generation TKI lorlatinib, as it has been shown efficacy in patients, who failed one or more second-generation TKIs [12]. However, this remained unsuccessful, which is in line with previous findings, that lorlatinib shows less efficacy in patients without *ALK* mutations [12]. Due to this short period (two months) under lorlatinib, we do not believe that lorlatinib treatment induced further resistance mutations in our patient. As the inactivating *TP53* mutation temporarily presented in a liquid biopsy but not in liver tissue, we rather assumed the *KRAS* mutation, which was already detected before lorlatinib treatment both in liquid biopsy and liver tissue, to be the molecular driver of this hepatic tumor progression. Therefore, we stopped TKI treatment and subsequently started combined chemoimmunotherapy and antiangiogenetic treatment according to the Impower150 protocol [16]. For the subgroup of patients with baseline liver metastases, this trial protocol showed improved median overall survival (OS) [23]. Also in our patient, this regimen was clinically beneficial. After completion of four cycles stable disease was reached. Moreover, the *KRAS*-mutant clone disappeared from liquid biopsies after two cycles together with clinical improvement. However, this benefit lasted only a short period, as the tumor extensively progressed after four cycles of maintenance therapy. Follow-up liquid biopsies revealed no further on- and off-target resistance mutations (Table 1). In contrast to the remarkable five-year OS rate of 62,5% for patients with advanced *ALK*-positive NSCLC first-line treated with alectinib [24], the patient in this case report had a rather short survival. Although the Impower150 protocol has been shown to be beneficial for patients with liver metastases [23], the presence of liver metastases is still considered a negative prognostic marker in NSCLC [25] and in this patient, disease progression was related exclusively to the liver. As there was no detectable cerebral tumor progression, this did not impact on survival. Furthermore, the value of immunotherapy in the subgroup of *ALK*-positive NSCLC is yet unclear. The Impower150 trial was the first trial to show benefit of adding atezolizumab to chemotherapy in patients with *EGFR* or *ALK* mutation. However, the authors themselves assume that this might be explained by immunomodulatory effects of bevacizumab that augment the efficacy of atezolizumab [23]. For NSCLC patients with *EGFR* or *ALK* mutation previous trials did not show superiority of PD-L1 or PD-1 inhibitors compared to standard chemotherapy after failure of TKI therapy [26–28]. Further trials are needed as there are no RCTs yet that specifically focus on the value of immunotherapy in the subgroup of *ALK*-positive NSCLC, probably due to small patient numbers. In contrast, there are numerous RCTs that have shown the lacking benefit of immunotherapy in never smokers [29, 30]. The patient in this case report stopped smoking twenty years ago and had only a ten pack-year history of tobacco smoking. Thus, we assume that i) the baseline presence and progression of liver metastases, ii) the unknown value of immunotherapy in *ALK*-positive NSCLC as well as iii) it’s less efficacy in never smokers, explain why the patient in this case report had only a short-term benefit from CIT and progressed rapidly after maintenance therapy, resulting in patient’s death.

**Table 1:** Off-target resistance mechanisms and treatment options in ALK-positive NSCLC

Off-target resistance mechanism	Treatment option	Literature
**EGFR activation**	Dual blockade of ALK and EGFR	Miyawaki, et al. 2017 [31] Tani, et al. 2016 [32]
**HER activation**	Dual blockade of ALK and EGFR	Tanizaki, et al. 2012 [33] Wilson, et al. 2015 [34]
**KIT amplification**	-	Katayama, et al. 2012 [35]
**MET amplification**	Dual blockade of ALK and MET	Dagogo-Jack, et al. 2020 [36]
**MEK reactivation**	Dual blockade of ALK and MEK	Hrustanovic, et al. 2015 [37] Shrestha, et al. 2020 [38]
**PIK3CA mutations**	-	Gainor, et al. 2016 [11] Crystal, et al. 2014 [39]
**IGF-1R activation**	Dual blockade of ALK and IGF-1R	Lovly, et al. 2014 [40]
**SRC activation**	Dual blockade of ALK and SRC	Crystal, et al. 2014 [39] Yoshida, et al. 2017 [41]
**TP53 mutation**	-	Gainor, et al. 2016 [11]
**KRAS mutations (excluding G12C)**	-	Doebele, et al. 2012 [20] Pailler, et al. 2019 [14]
**KRAS G12C mutation**	Sotorasib	McCoach, et al. 2018 [42] Hong, et al. 2020 [43]
**RET fusion**	Selpercatinib	Drilon, et al. 2020 [44] McCoach, et al. 2018 [42]

## CONCLUSIONS

In conclusion, this case report illustrates the development of two subsequent on- and off-target resistance mutations in a patient with *ALK*-fused NSCLC. We describe *ALK*-p.A1200_G1201delinsW as a so far undescribed mutation which confers resistance to alectinib but sensitivity to brigatinib. We provide further evidence that off-target *KRAS*-mutant clones i) give rise to resistance to ALK inhibitors in general and ii) might be addressed by chemoimmunotherapy. Therefore, our data highlights the importance of re-biopsies and molecular profiling to guide therapeutic decision-making according to clonal tumor evolution in *ALK*-positive NSCLC.
