# Addendum: Polyclonal on- and off-target resistance mutations in an EML4-ALK positive non-small cell lung cancer patient under ALK inhibition

**DOI:** 10.18632/oncotarget.28293

**Published:** 2022-11-02

**Authors:** Marcel Kemper, Georg Evers, Arik Bernard Schulze, Jan Sperveslage, Christoph Schülke, Georg Lenz, Thomas Herold, Wolfgang Hartmann, Hans-Ulrich Schildhaus, Annalen Bleckmann

**Affiliations:** ^1^Department of Medicine A, Hematology, Oncology, and Pneumology, University Hospital Muenster, 48149 Muenster, Germany; ^2^West German Cancer Center, Sites Muenster & Essen, 45147 Essen, Germany; ^3^Gerhard-Domagk-Institute of Pathology, University Hospital Muenster, 48149 Muenster, Germany; ^4^Institute of Clinical Radiology, University Hospital Muenster, 48149 Muenster, Germany; ^5^Institute of Pathology, University Hospital Essen, 45147 Essen, Germany; ^6^Department of Hematology/Medical Oncology, University Medical Center Goettingen, 37075 Goettingen, Germany; ^*^Authors share first authorship; ^#^Authors share last authorship

**This article has an addendum:** The authors obtained written informed consent from the patient to publish information and images.

Original article: Oncotarget. 2021; 12:1946–1952. 1946-1952. https://doi.org/10.18632/oncotarget.28062

